# Analysis of Macular Retinal Thickness and Microvascular System Changes in Children with Monocular Hyperopic Anisometropia and Severe Amblyopia

**DOI:** 10.1155/2022/9431044

**Published:** 2022-01-17

**Authors:** Lin-Lin Liu, Yu-Chuan Wang, Miao Cao, Fang Liu, Shuang Zhang, Jing Liu, Jin-Chang Liu, Lian-Feng Xie, Hui Wang

**Affiliations:** ^1^The Department of Ophthalmology of the 1st Affiliated Hospital, Gannan Medical University, Ganzhou, 341000 Jiangxi Province, China; ^2^Postgraduates at Gannan Medical University, Ganzhou, 341000 Jiangxi Province, China

## Abstract

**Objective:**

To study the changes of macular retinal thickness and microvascular system in children with monocular hyperopic anisometropia and severe amblyopia using optical coherence tomography angiography (OCTA) and to explore the value of OCTA in the diagnosis and treatment of amblyopia.

**Methods:**

Thirty-two children with monocular hyperopic anisometropia and severe amblyopia who were treated in the Department of Ophthalmology of the First Affiliated Hospital of Gannan Medical College from January 2020 to December 2020 were included in the study. Eyes with amblyopia (*n* = 32) served as the experimental group, and the contralateral healthy eyes (*n* = 32 eyes) served as the control group. All children underwent comprehensive ophthalmological examination including slit lamp, eye position, visual acuity, optometry, eye movement, intraocular pressure, ocular axis, and fundus examination to rule out organic lesions. Macular 6 mm × 6 mm scans were performed on both eyes of all subjects by the same experienced clinician using an OCTA instrument. After ImageJ processing, the vessel density, inner layer, and full-layer retinal thickness (RT) of superficial retinal capillary plexus (SCP) were obtained. All data were analyzed by SPSS21.0 software, and a paired *t*-test was used for comparison between groups. *P* < 0.05 was considered to indicate statistical significance.

**Results:**

The vessel densities of macular SCP in the amblyopia and control groups were 47.66 ± 2.36% and 50.37 ± 2.24% in the outer superior, 49.19 ± 2.64% and 51.44 ± 2.44% in the inner inferior, 49.63 ± 2.51% and 51.41 ± 3.03% in the outer inferior, and 45.56 ± 3.44% and 50.44 ± 3.52% in the outer temporal regions, respectively. The vessel density of macular SCP in the amblyopia group was significantly lower than that in contralateral healthy eyes in the outer superior, inner inferior, outer inferior, outer temporal, and central regions. There was no significant difference between the two groups in the inner superior, inner nasal, outer nasal, and inner temporal regions. The macular RT in the amblyopia group and the control group is 90.38 ± 6.09 *μ*m and 87.56 ± 5.55 *μ*m in the outer temporal, respectively. The RT in the macular inner layer in the outer temporal region of the amblyopia group was thicker than that of the control group (*P* < 0.05). There was no significant difference in the other eight regions between the two groups. The whole macular RT in the amblyopia group was thicker than that in the control group in nine regions, and the central area of macular RT in the amblyopia and control groups was 229.06 ± 6.70 *μ*m and 214.50 ± 10.36 *μ*m, respectively.

**Conclusion:**

The OCTA results showed the overall RT of macula in 9 areas in the amblyopia group was thicker than that in the control group, which could show that the macular retinal thickness can be a potential way to distinguish the children with monocular hyperopic anisometropia and severe amblyopia.

## 1. Introduction

Amblyopia is a common ophthalmic disease in children. Although eye examination does not show any organic lesion, the corrected visual acuity cannot reach the normal level. During the critical period of visual development, children are prone to amblyopia due to binocular abnormal action or form stripping. The incidence of amblyopia in China is 2–4% [1]. In recent years, the prevention and treatment of strabismus amblyopia in China has made steady progress, and remarkable achievements have been made in clinical diagnosis, treatment, epidemiological investigation, and early screening, but the specific pathogenesis of amblyopia is still under continuous exploration and research.

Optical coherence tomography angiography (OCTA) is a new ophthalmological imaging method, which is applied to the vascular imaging of the retina, choroid, and optic nerve. Compared with traditional fundus angiography, OCTA has the advantages of being noninvasive and fast, offers high-resolution and three-dimensional imaging, and can more accurately measure the size of capillary non-perfusion area and neovascularization [2]. The application potential of OCTA has attracted the attention of several ophthalmologists, but there are few studies on fundus thickness and microcirculation changes in children with amblyopia. We analyzed the changes of macular retinal thickness and microvascular system in children with monocular hyperopic anisometropia and severe amblyopia.

## 2. Materials and Methods

### 2.1. General Information

Thirty-two children with monocular hyperopic anisometropia and severe amblyopia who were treated in the Department of Ophthalmology of the First Affiliated Hospital of Gannan Medical College from January 2020 to December 2020 were included in the study. Eyes with amblyopia (*n* = 32) served as the experimental group, and the contralateral healthy eyes (*n* = 32) served as the control group. The following inclusion criteria were set in accordance with the consensus of amblyopic diagnostic experts in 2021 [3]: (1) children with monocular hyperopic anisometropic amblyopia and normal contralateral eyes; (2) severe amblyopia—best-corrected visual acuity ≤ 0.2; and (3) no organic eye lesions and systemic diseases. The exclusion criteria were as follows: (1) systemic and ocular diseases that cause changes in fundus microcirculation; (2) patients who were unable to cooperate with the examination due to various reasons; and (3) those with other eye diseases such as strabismus, ptosis, and cataract. This study was conducted in accordance with the tenets of the Helsinki Declaration, and the study was explained to the children and their parents/legal guardians; the latter provided written consent on behalf of the children. This study was approved by the Ethics Committee of the First Affiliated Hospital of Gannan Medical College.

### 2.2. Methods

#### 2.2.1. Routine Inspection

All selected patients underwent comprehensive ophthalmologic examination including slit lamp, eye position, visual acuity, optometry, eye movement, intraocular pressure, ocular axis, and fundus examination; all kinds of organic lesions were excluded. Moreover, the general indices of all candidates were recorded and analyzed, including age, sex, visual acuity, intraocular pressure, equivalent spherical optometry, and axial length. Objective optometry under the condition of ciliary paralysis was performed according to the expert consensus on the prevention and treatment of amblyopia in children (2021 Chinese Edition).

#### 2.2.2. OCTA Check

**Figure 1:**
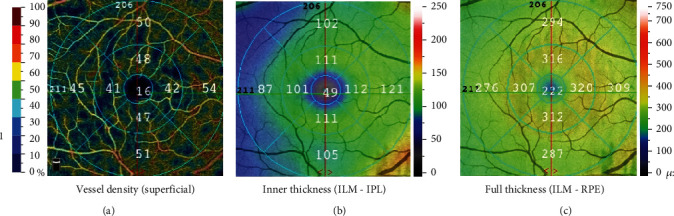
(a) Superficial vessel density of macula. (b) Inner thickness of macula, defined as the distance between the internal limiting membrane (ILM) and inner plexiform layer (IPL). (c) Full thickness of macula, defined as the distance from the ILM to the retinal pigment epithelium (RPE). The parafoveal and the foveal rim were divided into the upper, lower, nasal, and temporal quadrants. The macula was divided into nine regions, and their thickness values were displayed, respectively.

All included children were examined by the same experienced doctor for OCTA examination. The examination procedures were fully explained to the children at the start, and their cooperation was obtained. In this study, all subjects were performed by the AngioVue OCTA tester (OPTOVUE, USA), and ImageJ software was used to analyze all data. In this study, OCTA adopted a 6 mm × 6 mm macular scanning mode and Early Treatment Diabetic Retinopathy Study (ETDRS) zoning. The OCTA macular scanning mode could automatically scan macular superficial retinal vascular plexus (SCP) and deep retinal vascular plexus (DCP). The vessel density of SCP, macular inner retinal thickness (RT), and full-layer RT were detected and analyzed. The scanning area of SCP images 10 *μ*m from the internal limiting membrane (ILM) to the inner plexiform layer (IPL). The thickness of the inner layer of the retina is the distance from ILM to IPL, and the thickness of the whole layer is the distance from the ILM to the retinal pigment epithelium (RPE). The OCTA macular scan divides the macula into three circles: central fovea (diameter: 1 mm), perifovea (diameter: 1–3 mm ring), and central concave edge (diameter of the central ring: 3–6 mm). The paracavity and central concave edge were divided into the upper, lower, nasal, and temporal quadrants, which are subsequently divided into nine regions (Figure 1): the inner superior (IS), outer superior (OS), inner nasal (IN), outer nasal (ON), inner inferior (II), outer inferior (OI), inner temporal (IT), outer temporal (OT), and central (C) regions.

### 2.3. Statistical Methods

SPSS21.0 software (IBM Corporation, Armonk, NY, USA) was used for statistical analysis, and the quantitative data in accordance with normal distribution were expressed as the mean ± standard deviation (SD). The retinal SCP vessel density and the inner layer and full-thickness RT between amblyopic eyes and control eyes were compared by a paired *t*-test; *P* < 0.05 was considered to indicate statistically significant differences between the two groups.

## 3. Results

### 3.1. Basic Information of Patients

This study included 32 children (64 eyes, 18 male and 14 female) with monocular hyperopic anisometropia and severe amblyopia. Thirty-two eyes with amblyopia were included in the study group, and 32 contralateral healthy eyes were included in the control group. The mean age of the children was 6.2 ± 3.7 years. The homologous paired *t*-test was used in this study, which had strong balance and comparability, and there was no need to compare age and sex. In this study, the best-corrected visual acuity (BCVA), intraocular pressure (IOP), spherical equivalent (SE), and ocular axis were compared between eyes with amblyopia and healthy eyes. The results are shown in Table 1. There was no significant difference in IOP between the two groups, but there were significant intergroup differences in BCVA, SE, and ocular axis.

### 3.2. Vessel Density of SCP in Each Group

**Figure 2:**
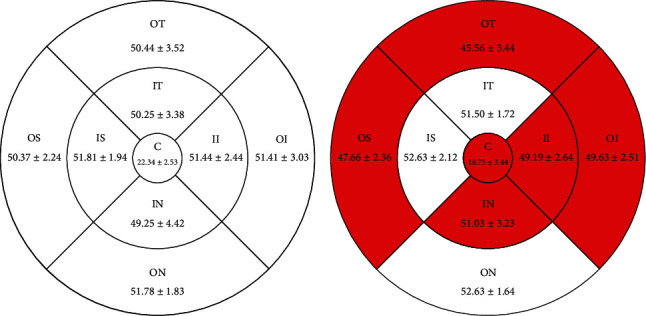
Results of superficial vessel density at different locations between amblyopia eye and healthy eye control (mean ± SD).

The vessel density of macular OS, II, OI, OT, and SCP in amblyopic eyes was lower than that in the control eyes (*P* < 0.05), but there were no significant intergroup differences with respect to macular IS, IN, ON, and IT. The results are shown in Table 2 and Figure 2.

### 3.3. Retinal Thickness of Macular Inner Layer Retina and of Whole Retina in Each Group

**Figure 3:**
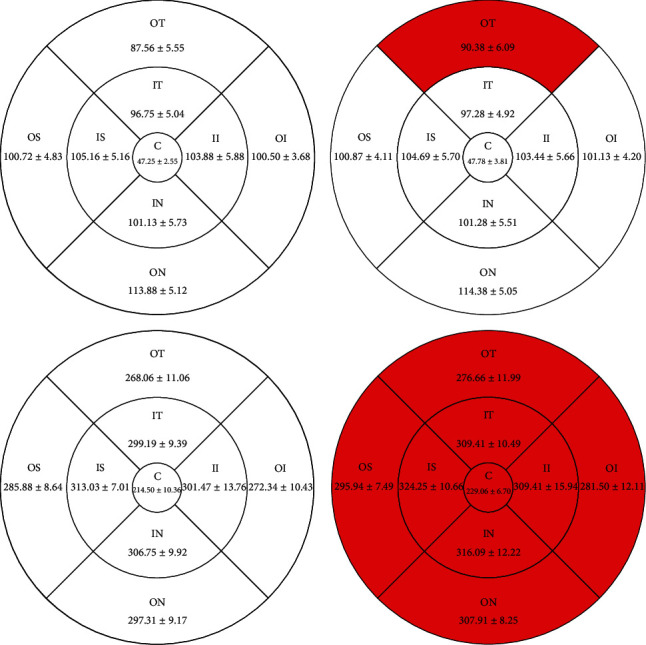
Results of macular retinal thickness at different locations between amblyopia eye and healthy eye controls (mean ± SD).

The RT in the inner layer of macular OT in amblyopic eyes was thicker than that in the control eyes (*P* < 0.05), but there were no significant differences in other areas between the two groups. The whole RT in the nine macular regions of amblyopic eyes was thicker than that of the control eyes (*P* < 0.05). The results are shown in Table 3 and Figure 3.

## 4. Discussion

In the past, OCT was usually used to study retinal thickness, but OCT cannot detect changes to the retinal microcirculatory system. With the continuous development of medical imaging technology, magnetic resonance imaging (MRI), functional MRI (fMRI), OCT, and OCTA are now commonly used to study amblyopia. In particular, OCTA technology has recently emerged and played a revolutionary role in the study of retinal microvascular system [4]. OCTA can minimally, simply, and conveniently display the retinal morphology in great detail and can detect the vessel signals of the retinal microvascular system and the tiny changes to RT. Further, OCTA's application on retinal microvascular system research has aroused the interest of multiple scholars. In related research on the retina of patients with amblyopia, Nishikawa et al. [5] scanned the macular area of OCTA in 22 children with monocular amblyopia. It was seen that the vessel density of the central fovea and accessory central fovea in amblyopic eyes was lower than that in the healthy contralateral eyes, and the central foveal avascular area in amblyopic eyes was significantly smaller than that in the contralateral eyes. Some researchers [6–11] found that the vascular density of SCP and DCP decreased in amblyopic eyes. However, other studies [12, 13] reported that the blood vessel density of SCP and DCP in amblyopic eyes remained unchanged compared with healthy eyes.

Recent studies [14] showed that the vessel density of SCP in amblyopic eyes was lower than that in the control group. Huynh et al. [15–17] found that the macular thickness in amblyopic eyes was thicker than that in contralateral eyes. Li et al. [18] showed that the process of amblyopia may involve changes to the retinal microcirculatory system, and the macular fovea of amblyopic eyes was thicker than that of normal eyes. Atakan et al. [9, 19, 20] found no significant change between the amblyopic and control groups. Khan et al. [21] found the following results in their study on the relationship between retinal microvessel density and ocular axis in the macular region: the longer the eye axis, the smaller the superficial perfusion area and blood vessel density, and the retinal macular vessel density is inversely proportional to the size of ocular axis. Some previous studies [22–24] reported a negative correlation between AL and central foveal macular thickness in children with healthy eyes, although another study did not find this correlation [25]. Therefore, conclusions about the retinal microvessels and thickness in patients with amblyopia are fairly inconsistent.

Our results showed that in the study of retinal SCP vessel density in children with monocular hyperopic anisometropic amblyopia, the SCP vessel density in amblyopic eyes was significantly lower than that in contralateral healthy eyes in the macular OS, II, OI, OT, and C regions; however, there were no significant intergroup differences in SCP vessel density in the macular IS, IN, ON, and IT regions. This study also found that the inner layer RT of macular OT in amblyopic eyes was significantly higher than that in the contralateral healthy eyes, while the inner layer RT in the remaining eight regions only showed minor changes, but these changes were not significantly different between the two groups. The full-layer RT in nine macular regions of amblyopic eyes was thicker than that of contralateral healthy eyes, and the difference was statistically significant. At present, most studies on retinal vessel density and RT of amblyopia have not been classified according to the type of amblyopia and refractive state. The etiology of amblyopia and refractive state may affect the retinal vessel density and RT.

In this study, OCTA was used to analyze the retina of children with severe amblyopia caused by monocular hypermetropia, and the results were more comparable, which may be why our results are distinct from other studies. At present, most researchers study the average thickness and mean vessel density of the macular fovea, and few scholars subdivide the macular region into nine regions for comparative analyses. The decrease of vessel density in amblyopic eyes may indicate that these eyes need less nutrition to receive retinal artery blood supply, resulting in abnormal macular fovea development and retardation in amblyopic eyes. Because the nasal side of the retinal vascular system develops earlier than the temporal side and the microcirculation in different regions is not uniformed, we thought it was more meaningful to subdivide the macula into nine regions in this study.

Our study has some limitations. First, the sample size of the study is small. Second, the data accuracy was affected by the poor matching degree detected by OCTA in children. Third, the control group of myopic anisometropic amblyopia and other types of amblyopia have not been established at the same time. Fourth, the effects of equivalent spherical lens and ocular axis on retinal vessel density and retinal thickness cannot be excluded. Last, the current OCTA technique cannot detect the vessel velocity of retinal microcirculation and the elasticity and diameter of the microvessels.

In conclusion, the emergence of OCTA technology has significant advantages to study the retinal microvascular system changes. The clinical application of OCTA may have a certain exploration value in the pathogenesis, diagnosis, and treatment of amblyopia and is expected to play an important role in its diagnosis and treatment.

## Figures and Tables

**Table 1:** Characteristics of eyes with amblyopia and healthy eyes.

Characteristic	Amblyopia eyes (*n* = 32)	Healthy eyes (*n* = 32)	*t*/*χ*^2^	*P* value
Best-corrected visual acuity	0.12 ± 0.06	1.04 ± 0.09	45.28	<0.001
Mean intraocular pressure (mmHg)	16.72 ± 1.89	16.56 ± 1.90	0.407	0.687
Spherical equivalent refraction (D)	6.05 ± 1.90	0.31 ± 0.51	15.55	<0.001
Length of optic axis (mm)	23.00 ± 0.34	23.45 ± 0.20	7.219	<0.001

**Table 2:** Comparison of superficial vessel density at different locations between eyes with amblyopia eye and healthy eyes.

Location (%, mean ± SD)	Amblyopia eyes (*n* = 32) (%)	Healthy eyes (*n* = 32) (%)	*t* value	*P* value
IS	52.63 ± 2.12	51.81 ± 1.94	1.946	0.061
OS	47.66 ± 2.36	50.37 ± 2.24	5.988	<0.001
IN	51.03 ± 3.23	49.25 ± 4.42	2.063	0.048
ON	52.63 ± 1.64	51.78 ± 1.83	1.851	0.074
II	49.19 ± 2.64	51.44 ± 2.44	4.313	<0.001
OI	49.63 ± 2.51	51.41 ± 3.03	3.368	0.002
IT	51.50 ± 1.72	50.25 ± 3.38	1.816	0.079
OT	45.56 ± 3.44	50.44 ± 3.52	12.158	<0.001
C	18.75 ± 3.44	22.34 ± 2.53	11.950	<0.001

SD: standard deviation; IS: inner superior; OS: outer superior; IN: inner nasal; ON: outer nasal; II: inner inferior; OI: outer inferior; IT: inner temporal; OT: outer temporal; C: central.

**Table 3:** Comparison of macular retinal thickness at different locations between amblyopia eye and healthy eye controls.

Location	Amblyopia eyes (*n* = 32) (*μ*m)	Healthy eyes (*n* = 32) (*μ*m)	*t* value	*P* value
Macular inner retinal thickness (*μ*m), mean ± SD				
IS	104.69 ± 5.70	105.16 ± 5.16	1.305	0.201
OS	100.87 ± 4.11	100.72 ± 4.83	0.181	0.857
IN	101.28 ± 5.51	101.13 ± 5.73	0.469	0.643
ON	114.38 ± 5.05	113.88 ± 5.12	1.806	0.081
II	103.44 ± 5.66	103.88 ± 5.88	1.238	0.225
OI	101.13 ± 4.20	100.50 ± 3.68	1.341	0.190
IT	97.28 ± 4.92	96.75 ± 5.04	1.848	0.074
OT	90.38 ± 6.09	87.56 ± 5.55	8.057	<0.001
C	47.78 ± 3.81	47.25 ± 2.55	1.240	0.224
Macular full retinal thickness (*μ*m), mean ± SD				
IS	324.25 ± 10.66	313.03 ± 7.01	8.965	<0.001
OS	295.94 ± 7.49	285.88 ± 8.64	8.790	<0.001
IN	316.09 ± 12.22	306.75 ± 9.92	7.550	<0.001
ON	307.91 ± 8.25	297.31 ± 9.17	12.386	<0.001
II	309.41 ± 15.94	301.47 ± 13.76	6.711	<0.001
OI	281.50 ± 12.11	272.34 ± 10.43	7.282	<0.001
IT	309.41 ± 10.49	299.19 ± 9.39	7.660	<0.001
OT	276.66 ± 11.99	268.06 ± 11.06	7.313	<0.001
C	229.06 ± 6.70	214.50 ± 10.36	8.185	<0.001

SD: standard deviation; IS: inner superior; OS: outer superior; IN: inner nasal; ON: outer nasal; II: inner inferior; OI: outer inferior; IT: inner temporal; OT: outer temporal; C: central.

## Data Availability

The data can be found at OCTA Article original data of figure files.
